# Extracorporeal cardiac shock wave therapy modulates post-infarction neovascularization via CAC-mediated pro-angiogenic effects and modulation of the S100A4/CSF2/FOXO1 protein network

**DOI:** 10.3389/fcvm.2026.1839337

**Published:** 2026-06-12

**Authors:** Yang Li, Hao Li, Chen Liu, Xiaoying Zeng, Xin Tian, Wenwen Hu, Luqiao Wang, Baotong Hua, Ping Yang

**Affiliations:** 1 Cardiovascular Clinical Medicine Center, The First Affiliated Hospital of Kunming Medical University, Kunming, China; 2 Department of Radiology, Second People’s Hospital of Yunnan Province, Kunming, China; 3 School of Public Health, Kunming Medical University, Kunming, China

**Keywords:** cardiac shock wave therapy, circulating angiogenic cells, ischemic heart disease, myocardial repair, neovascularization

## Abstract

Ischemic heart disease (IHD) remains the leading cause of mortality worldwide, and the long-term benefits conferred by revascularization are limited. Cardiac shock wave therapy (CSWT) can promote neovascularization and attenuate myocardial injury, and has been applied in the treatment of IHD; its mechanism may involve Circulating Angiogenic cells (CACs) and their key proteins. This study aimed to explore the potential mechanism by which CSWT exerts effects on angiogenesis and attenuates structural damage following Acute Myocardial Infarction (AMI). Using *in vivo* and *in vitro* experiments combined with Olink proteomics, three core regulatory proteins involved in angiogenesis, namely S100A4, CSF2, and FOXO1, were screened. *In vitro*, optimized CSWT enhanced the migration and tube formation of rat bone marrow-derived CACs, as well as the expression of pro-angiogenic factors such as VEGF and HGF. *In vivo*, CSWT after AMI reduced infarct size, elevated the levels of CACs (CD34/CD133) and vascular markers (CD31/*α*-SMA), and modulated 18 differentially expressed proteins. Key regulatory factors S100A4, CSF2, and FOXO1 were identified by Olink proteomics. The expression levels of these proteins were verified by dual qPCR experiments *in vitro* and *in vivo*. The results reveal that CSWT modulates the S100A4/CSF2/FOXO1 network and activates CACs in AMI, suggesting a mechanistic basis for further translational evaluation of CSWT as a potential adjuvant therapy for IHD.

## Introduction

Ischemic heart disease (IHD) remains the leading global cause of mortality, characterized by a complex pathological progression involving cardiomyocyte loss, adverse remodeling, and eventual heart failure (1–5). Despite the efficacy of revascularization strategies like Percutaneous Coronary Intervention (PCI), a significant proportion of patients continue to face limited long-term prognostic benefits and a high risk of major adverse cardiovascular events (6). Consequently, identifying innovative strategies that promote myocardial repair and functional neovascularization is essential to address the unmet clinical needs in IHD management.

In this context, extracorporeal cardiac shock wave therapy (CSWT), a non-invasive emerging therapy that delivers low-energy shock waves to targeted myocardial regions to elicit beneficial biological effects, has shown promising therapeutic potential in clinical trials (7). Its core mechanisms encompass two primary aspects: first, the stimulation of angiogenesis to improve blood supply and oxygen delivery to ischemic myocardium; and second, the modulation of key signaling pathways to mitigate excessive inflammation and foster an anti-inflammatory milieu. This dual pro-angiogenic and anti-inflammatory effect creates a favorable microenvironment that supports structural preservation and tissue viability of the ischemic heart (8, 9). Early studies have indicated that a pivotal element of CSWT's angiogenic promotion is its effective mobilization and activation of circulating angiogenic cells (CACs), which in turn augment endogenous repair and neovascularization in ischemic myocardium (10).

CACs represent a heterogeneous population of cells in the peripheral blood, primarily functioning to detect and respond to signals of tissue ischemia or injury, mobilize to the damaged site, and contribute to vascular homeostasis maintenance as well as pathological repair and regeneration (11). The mechanical stimulation from CSWT induces the secretion of pro-angiogenic factors (e.g., VEGF, IL-8), which recruit CACs from the bone marrow into circulation and guide their migration to ischemic myocardial tissue (12). Moreover, CSWT activates the PI3 K/Akt/eNOS signaling pathway, thereby enhancing the function of endothelial progenitor cells (EPCs)—a key CACs subtype involved in vascular repair—following hypoxic injury (13). These augmented EPCs substantially contribute to myocardial repair processes. Collectively, the fundamental mechanism driving the robust pro-angiogenic capability of CACs hinges on the secretion of and response to pro-angiogenic factors.

While classical pro-angiogenic factors are central to revascularization, the functional activation of CACs is stringently governed by a broader and more complex molecular microenvironment. Our previous work confirmed that CSWT enhances cardiac repair via the PI3 K/Akt pathway (14, 15). However, the overarching protein networks driving this paracrine and angiogenic cascade under CSWT remain incompletely mapped. Emerging evidence highlights several critical, yet distinct, regulators in this context. For instance, Granulocyte-Macrophage Colony-Stimulating Factor (CSF2/GM-CSF) is a potent positive regulator that mobilizes progenitor cells and stimulates their homing to ischemic tissues via MAPK and PI3 K signaling (16). Conversely, Forkhead box O1 (FoxO1) acts as an intrinsic negative checkpoint for vascular endothelia, driving a strong anti-angiogenic phenotype in ischemic environments (17). Concurrently, S100 calcium-binding protein A4 (S100A4) is heavily implicated in adverse cardiac remodeling and endothelial-to-mesenchymal transition, which can compromise functional vascular networks (18). The potential interplay between these contrasting regulatory nodes—CSF2 as a promoter, and FOXO1/S100A4 as inhibitors—represents a plausible mechanistic framework for CSWT's efficacy.

Therefore, to comprehensively identify the key regulatory networks underlying CSWT, we employed Olink proteomics to screen for differentially expressed proteins. Based on this proteomic screening and the established biological roles of the aforementioned factors, we aimed to investigate whether cardiac shock wave therapy modulates neovascularization and attenuates structural damage in acute infarction by regulating this specific protein axis. Specifically, we hypothesized that CSWT is associated with the modulation of the S100A4/CSF2/FOXO1 network—including the targeted downregulation of FOXO1 and S100A4, and the upregulation of CSF2—to facilitate the paracrine activation of CACs. To test this exploratory hypothesis and further understand the potential underlying mechanisms, we conducted the following studies: *in vitro* shock wave treatment of CACs under ischemic conditions, *in vivo* validation using a myocardial infarction (MI) model, and a comprehensive evaluation integrating proteomic, molecular biology, and functional analyses.

### Subsection

1. Rat model construction

Thirty male Sprague-Dawley rats [aged 5–6 weeks; supplied by Henan Sibeifu Biotechnology Co., Ltd.; Animal Production License No. SCXK (Yu) 2020-0005; Animal Use License No. SYXK (Dian) K2020-0006] were utilized for the *in vivo* study. The MI model was established via ligation of the left anterior descending (LAD) coronary artery as previously describe (19). Briefly, rats were anesthetized with 2% pentobarbital sodium (10 mL/kg, i.p.), intubated, and ventilated (80 breaths/min). Following a left thoracotomy at the 3rd–4th intercostal space, the LAD was ligated with 6-0 silk sutures. Successful induction was confirmed by visual myocardial pallor and ST-segment elevation on the electrocardiogram. Postoperative analgesia was provided via subcutaneous buprenorphine (0.05 mg/kg) for 72 h.

Rats with failed MI induction or perioperative death were excluded. The remaining successfully modeled animals were randomly divided via a computer-generated random sequence (SPSS Statistics, version 26.0; IBM Corp., Armonk, NY, USA), with a consistent final sample size of *n* = 6 in each group for subsequent experiments. The experimental groups included: (1) Control group, (2) Acute myocardial infarction (MI) group, and (3) MI + cardiac shock wave therapy (MI + CSWT) group. For the MI + CSWT group, after identical MI induction, rats received cardiac shock wave therapy (MODULITH SLC; Storz Medical AG, Switzerland) on postoperative days 5, 7, 9, 11, 13, 15, 17, 19, and 21. Under isoflurane anesthesia (induction: 5% vol in O₂ at 2 L/min; maintenance: 1.5% vol), the shaved animals underwent ECG-monitored therapy (target heart rate: 400 ± 50 bpm). An integrated ultrasound probe (ALOKA SSD-900) was used to target the anterior apical wall of the left ventricle. Shock waves (energy flux density: 0.09 mJ/mm^2^) were delivered to a 9-point matrix (200 shocks per point; total: 1800 shocks) using fine-adjustment controls (±3 units; upper knob: 6° per unit for angular adjustment; lower knob: 2.5 mm per unit for focal displacement), with contact maintained via a water cushion. All rats were euthanized via intraperitoneal injection of 2% sodium pentobarbital 24 h after the final treatment for tissue collection. All animal experiments were conducted in strict accordance with the 3R principles (replacement, reduction, and refinement) and were approved by the Animal Experiment Ethics Review Committee of Kunming Medical University (Approval No. 1977900331; approved on July 11, 2023).

2. Isolation and culture of CACs

Five male Sprague-Dawley rats [aged 5–6 weeks; supplied by Henan Specific Bio-technology Co., Ltd.; Production License No. SCXK (Henan) 2020-0005; Use License No. SYXK (Yunnan) K2020-0006] were anesthetized with an overdose of 2% sodium pentobarbital (150 mg/kg, i.p.) and monitored for cardiac and respiratory arrest to confirm the absence of reflexes. Following confirmation, the rats' fur was wetted with 75% alcohol for disinfection. The skin along the thighs was incised with scissors to expose and remove the muscles, allowing extraction of the bilateral tibiae. The excised tibiae were placed in a Petri dish containing phosphate-buffered saline (PBS) supplemented with 5% penicillin-streptomycin (double antibiotic) to remove adhering impurities and blood. The cleaned tibiae were then transferred to a fresh Petri dish with PBS, and the bone marrow was flushed out using a pipette to aspirate PBS through the medullary cavity, followed by thorough mixing via pipetting. Six milliliters of Histopaque-1083 (Sigma, Cat. No. 10831) were added to a 15 mL centrifuge tube, and the bone marrow suspension was gently layered on top along the tube wall using a pipette. The sample was centrifuged at 500 × g for 15 min, after which the white mononuclear cell layer was collected into a new centrifuge tube, mixed with 3 mL PBS, and centrifuged again at 250 × g for 5 min. The supernatant was discarded, and the cell pellet was resuspended in 1 mL complete medium [89% EBM-2 medium+10% fetal bovine serum (FBS) + 1% penicillin-streptomycin]. The resuspended cells were transferred to a fibronectin-precoated T25 flask containing an additional 4 mL of complete medium, mixed gently in a figure-eight motion, and incubated at 37 °C with 5% CO₂. Cells were observed under an inverted microscope; when approximately 60% adherence was achieved (with some floating cells and debris), the medium was discarded, and 5 mL fresh complete medium was added to continue incubation. Upon reaching 90% confluence, cells were subcultured for further experiments.

3. Flow cytometric analysis of CACs surface markers

CACs were detached using 0.25% trypsin (Biosharp, Cat. No. BL526A) at 37 °C for 3 min, and digestion was terminated by adding DMEM/F-12 medium supplemented with 10% FBS. The cells were centrifuged at 300 × g for 5 min, the supernatant was discarded, and the cell pellet was collected. The pellet was washed once with 50 μL of 1 × PBS, gently resuspended, and counted. Per sample tube, 1 × 10⁵ cells were aliquoted and resuspended in 100 μL of 1 × PBS. Samples were incubated with 5 μL of anti-CD133 antibody (Invitrogen, Cat. No. 12-1331-80) and 5 μL of anti-CD34 antibody (Invitrogen, Cat. No. 11-0341-81) at 4 °C for 30 min in the dark. Each tube was then supplemented with 400 μL of 1 × PBS and filtered. Stained samples were analyzed on a BD FACSCalibur flow cytometer, acquiring 10,000 events per sample. Data were processed using FlowJo software (v10.8.1) to determine the percentage of cells expressing the markers. Rigorous definition of the CAC population was achieved through a sequential gating strategy. Cell debris and mature circulating leukocytes were initially excluded by their forward and side scatter (FSC/SSC) characteristics. The proportion of CD34⁺CD133⁺ double-positive cells—a canonical immunophenotype for primitive, uncommitted progenitor cells—was subsequently quantified, thereby confirming the phenotypic purity of the isolated CACs (Supplementary Figure S1).

4. Shock wave intervention

Frozen CACs vials were thawed in a 37 °C water bath for 1 min, surface-sterilized with 75% ethanol, and transferred to 15 mL centrifuge tubes containing 9 mL complete medium [89% endothelial cell basal medium (PriMed-iCell-002) + 10% FBS+1% penicillin/streptomycin]. Cells were centrifuged at 200 × g (1,000 rpm) for 5 min, the supernatant was discarded, and the pellet was resuspended in 1 mL fresh complete medium. The cell suspension was evenly distributed into three T25 flasks, each supplemented with 4 mL complete medium. Upon reaching 80% confluence, CACs were washed twice with PBS, detached using 0.25% trypsin (Meron Bio, Cat. No. MA0233) until cell rounding was observed, and digestion was neutralized with complete medium. Cells were gently pipetted to form a single-cell suspension, centrifuged at 200 × g for 5 min, and subcultured. For shock wave treatment, CACs were exposed to energy flux densities of 0.03, 0.06, 0.09, 0.12, 0.15, and 0.18 mJ/mm^2^ at a frequency of 1 Hz using the MODULITH SLC device (Storz Medical AG, Switzerland). The probe-mounted container was adjusted to a water cushion height of 6.5 cm to ensure full contact with the container base, tilted upward at 30°–45°, and filled with distilled water. Individual T25 flasks were positioned such that the cell monolayer faced the water interface. Each flask received 500 shocks, with successful energy delivery confirmed by acoustic emission.

5. Co-culture of RUVEC cells with CACs cells

Co-cultures were established using Transwell chambers (0.4 μm pore size; Corning, Cat. No. 3412, USA). Rat umbilical vein endothelial cells (RUVECs; Novozymes Procell, Cat. No. CP-R232) were seeded in the lower chamber of a 6-well plate with 2 mL EBM-2 medium, while shock wave-treated CACs were seeded in the upper chamber. After 24 h of co-culture, plates were removed from the incubator, supernatants were discarded, and cells were rinsed twice with PBS. Cells were detached using 0.25% trypsin (Biosharp, Cat. No. BL526A) for 1–2 min, with digestion terminated by adding an equal volume of complete medium. Detached cells were collected by centrifugation at 200 × g (1,000 rpm) for 5 min.

6. Cell proliferation was assessed using the CCK-8 assay

Shock wave-treated CACs were seeded in 96-well plates at a density of 3,000 cells per well and incubated at 37 °C for 24 h. Subsequently, 10 μL of CCK-8 reagent (Elabscience, Cat. No. E-CK-A362) was added to each well. Plates were gently agitated for 60 s to ensure uniform mixing and returned to the 37 °C incubator for 2 h. Absorbance was measured at 450 nm using a microplate reader (BioTek, ELx800). For data processing, blank control wells (containing medium and CCK-8 reagent only) were used for background correction. Experimental optical density (OD) values were calculated as the measured value minus the mean blank control OD. Cell proliferation rate (%) was determined as (mean experimental OD/mean control OD) × 100%. Proliferation curves were generated with time on the *x*-axis and OD values on the *y*-axis, where higher OD values indicated greater viable cell numbers and enhanced proliferative capacity.

7. Transwell cell migration

Cell migration was evaluated using Transwell chambers equipped with 8.0 μm pore membranes (Beijing Lanjieke Science and Technology, Cat. No. 12100089). Shock wave-treated CACs were resuspended in serum-free medium at a density of 5 × 10⁵ cells/mL, and 100 μL aliquots were seeded into the upper chambers. The lower chambers were filled with 600 μL EBM-2 medium supplemented with 10% FBS. Following a 3-h incubation at 37 °C with 5% CO₂, migrated cells on the undersurface of the membranes were fixed with 4% methanol for 15 min, stained with 0.1% crystal violet for 5 min, and rinsed three times with distilled water. Membranes were air-dried, mounted on slides, and five random fields per membrane were imaged under an inverted microscope at 100 × magnification. Migrated cells were quantified using ImageJ software by an independent investigator blinded to the experimental conditions.

8. Tube formation assay

Tube formation assays were performed as follows: Matrigel matrix (ABW, Cat. No. 827045) was thawed overnight at 4 °C, avoiding freeze-thaw cycles. For matrix coating (conducted on ice to prevent premature polymerization), 24-well plates and pipette tips were pre-chilled to 4 °C. Each well was coated with 60 μL Matrigel to achieve uniform thickness without bubbles, and plates were incubated at 37 °C for 30 min to allow complete polymerization. RUVECs in the logarithmic growth phase were trypsinized, resuspended in culture medium, and counted. Cell density was adjusted to 1–2 × 10⁵ cells/mL. Subsequently, 100 μL of cell suspension was added to each well of the 24-well plate, supplemented with 400 μL medium, and plates were gently rocked for even distribution. After 12 h of incubation at 37 °C with 5% CO₂, lumen structure formation time, tubular structure length, number of branch points, and network integrity were assessed using an inverted microscope at 4 × and 100 × magnifications. ImageJ software was used to quantify the number of vascular branches, trunk length, and nodes in a blinded manner to eliminate observer bias.

9. Immunohistochemical (IHC) staining detection

Cardiac tissues were fixed overnight in 4% paraformaldehyde (Koufu, CNAB035-Q), dehydrated through an ethanol gradient (Chengdu Cologne Chemical Co., Ltd., GB/T678-2002), cleared in xylene (Xilong Chemical Co., Ltd., 33535), embedded in paraffin wax, and sectioned at 3 μm thickness for immunohistochemical staining. Tissue sections were baked at 64 °C for 1 h in a thermostat (Shaoxing Isofai Instrument Co., Ltd., 303-2). Subsequently, sections were deparaffinized in xylene, rehydrated through graded alcohols, and subjected to antigen retrieval in citrate buffer (Xavier, G0001-1L). Endogenous peroxidase activity was quenched by incubation in 3% H₂O₂ for 20 min at room temperature. Sections were then blocked with 5% bovine serum albumin (BSA) at 37 °C for 30 min. Primary antibodies against VEGF (Bioss, bs-1313R, 1:100), HGF (Affinity, DF6326, 1:50), bFGF (Affinity, DF6038, 1:50), and SDF-1 (HUABIO, ER1902-35, 1:50) were diluted in 2% BSA according to the manufacturers' instructions. The diluted primary antibodies were applied to the sections and incubated. Following primary antibody incubation, sections were incubated with an enhanced enzyme-labelled sheep anti-mouse IgG polymer (Servicebio, GB23303) at 37 °C for 20 min. After three washes with PBS (Xavier, G0002-2L), DAB staining solution (Zhongsui Jinqiao, ZLI-9019) was applied dropwise. Upon development of visible staining, sections were washed three times with PBS, counterstained with hematoxylin (Xavier, G1004-100 mL) for 5 min, differentiated in acid alcohol, blued in water, dehydrated through graded alcohols, cleared in xylene, and mounted with neutral gum (Xavier, WG10004160). Images were captured at 200x/400× magnification using an Olympus BX51 microscope, with five random fields of view analyzed per section. Integrated optical density (IOD) values were quantified using ImageJ software by two independent observers blinded to the treatment allocations.

10. Immunofluorescence (IF) detection

Tissues were fixed overnight in 4% paraformaldehyde (Koufu, CNAB035-Q), dehydrated through an ethanol gradient (Chengdu Cologne Chemical Co., Ltd., GB/T678-2002), cleared in xylene (Xilong Chemical Co., Ltd., 33535), embedded in paraffin, and sectioned at 3 μm thickness for immunofluorescence (IF) staining. Sections were baked at 64 °C for 1 h in a thermostat (Shaoxing Isofai Instrument Co., Ltd., 303-2). Subsequently, sections were deparaffinized in xylene, rehydrated through graded alcohols, and subjected to antigen retrieval in sodium citrate buffer (Xavier, G0001-1L). To enhance antibody permeability, sections were incubated in permeabilization solution (40 mL PBS, 400 μL 30% H₂O₂, 120 μL Triton X-100) at room temperature for 20 min. Non-specific binding was blocked with 5% bovine serum albumin (BSA; Gibco) at 37 °C for 30 min. After three 5-min washes with PBS (Xavier, G0002-2L), primary antibodies against CD34 (Affinity, DF6139, 1:100), CD133 (Affinity, AF512, 1:100), CD31 (Proteintech, 28083-1-AP, 1:100), and *α*-SMA (CST, 19245S, 1:100) diluted in 2% BSA were applied and incubated at 4 °C overnight. Sections were then incubated with secondary antibodies [Goat anti-Rabbit IgG H&L (Alexa Fluor® 488; abcam, ab150077) and Goat anti-Rabbit IgG H&L (Alexa Fluor® 555; abcam, ab150078)] at 37 °C for 20 min. Nuclei were stained with DAPI (Ex:330-380 nm, Em:420 nm; blue), while CD34 and CD133 were labeled with Alexa Fluor® 488 (green) and Alexa Fluor® 555 (red), respectively.

11. Western blot (WB) analysis

RUVEC cells were isolated from co-cultures of CACs/RUVEC and shock wave-treated CACs/RUVEC. Cells were lysed with 500 µL ice-cold RIPA buffer (Servicebio, G2002-30 mL) supplemented with protease inhibitors (Proteintech, PR20032) for 10 min at 4 °C, followed by centrifugation at 14,000 × g for 15 min. Protein concentrations were determined using a BCA assay kit (Beyotime, P0009). For each sample, 80 μg of protein was mixed with 20 μL 5 × loading buffer and denatured at 95 °C for 5 min. Proteins were separated by 10% SDS-PAGE (Solarbio, S1010) and transferred to PVDF membranes (Millipore, K2MA8350E). Membranes were blocked with 5% BSA (Solarbio, CR2302110) for 1 h at room temperature, then incubated overnight at 4 °C with primary antibodies against VEGF (Bioss, bs-1313R, 1:2000), HGF (Affinity, DF6326, 1:2000), bFGF (Affinity, DF6038, 1:2000), SDF-1 (HUABIO, ER1902-35, 1:500), and *β*-actin (Proteintech, 66009-1-Ig, 1:25,000). After washing, membranes were incubated for 40 min with HRP-conjugated secondary antibodies: goat anti-rabbit IgG (Servicebio, GB23303, 1:3000) for VEGF/HGF/bFGF/SDF-1, and goat anti-mouse IgG (Servicebio, GB23301, 1:5000) for *β*-actin. Protein bands were visualized using a high-sensitivity ECL kit (Affinity, KF8001) and imaged with a VILBER Chemiluminescence Imaging System. Band intensities were quantified using ImageJ software (v1.8.0.345) by an investigator blinded to the sample identities, with target protein expression normalized to *β*-actin (integrated density ratio=target protein/internal control).

12. Quantitative real-time PCR (qPCR) analysis was performed

Total RNA was extracted from tissues or cells using TRIzol reagent (Ambion, 15596-018CN). RNA concentration and purity were determined using a NanoDrop spectrophotometer. The SweScript First Strand cDNA Synthesis Kit (Servicebio) was used for reverse transcription. Briefly, a total of 2 μg of RNA was added to the reaction system, and reverse transcription was performed using a conventional PCR instrument (Bio-Rad PCR System) according to the manufacturer's instructions. The resulting cDNA was diluted 5–20-fold with RNase/DNase-free ddH₂O.

RT-qPCR was subsequently carried out on a CFX Connect Real-Time PCR System. The optimized two-step thermocycling conditions were as follows: 95 °C for 30 s (initial denaturation), followed by 40 cycles of 95 °C for 5 s (denaturation) and 60 °C for 30 s (annealing/extension). GAPDH was used as the internal control for normalization. The relative expression levels of target genes were calculated using the 2- △ △ Ct method. All primers used in this study (Table 1) were rigorously validated for specificity, as evidenced by a single discrete peak in the final melting curve analysis (60 °C to 95 °C) without non-specific amplification.

13. TTC staining

**Table 1:** qPCR primer design sequences.

Primer	Sequence
*Vegf* F	ATGCGGATCAAACCTCACCA
*Vegf* R	CTTGGCTTGTCACATACGCTC
*Hgf* F	CCCTATGCAGGACAGAAGAAGAG
*Hgf* R	TCTGATGCACCTGTTGGCAC
*Bfgf* F	AAAACCTGACCCGATCCCTC
*Bfgf* R	CGTGACGCAGCTCCTAAAGT
*Sdf-1* F	GACAAGTGTGCATTGACCCG
*Sdf-1* R	CAGAGCAGAGCGTACTGTGG
*S100a4* F	TCAGCACTTCCTCTCTCTTGG
*S100a4* R	GCTTCTGGAATGCAGCTTCG
*Csf2* F	CCACTACCAGACGAACTGCC
*Csf2* R	TCCTCATTTCTGGACCGGCT
*Foxo1* F	CACCTCTGCTGAATGACGGA
*Foxo1* R	GCAGATTGATCACCGGCTACT
Internal reference R- *Gapdh* F	GGCCGGAGACGAATGGAAATTA
Internal reference R-*Gapdh* F	CCAAATCCGTTCACACCGAC

Isolated hearts from control (*n* = 6), myocardial infarction (MI) model (*n* = 6), and MI model with cardiac shock wave therapy (CSWT) groups (*n* = 6) were transferred in ice-cold PBS (0-4℃) and flash-frozen at −20 °C for 30 min. Cardiac tissues were sectioned transversely into 2-mm thick slices. Sections were immersed in 1% TTC staining solution (Solarbio, 20250811) and incubated at 37℃ in a light-protected water bath for 30 min with gentle agitation every 5 min. After staining, slices were rinsed in PBS for 5 min and fixed in 10% neutral buffered formalin for 6 h. Digital images were acquired for each slice, with the caudal surface selected for analysis. Using ImageJ software, the infarct area (unstained region) and total left ventricular area were measured per slice by an investigator who was blinded to the group assignments. Infarct volume per slice was calculated as: (infarct area) × (slice thickness). Total infarct volume was determined by summing all slice volumes. Myocardial infarct size was expressed as a percentage of total left ventricular volume: (total infarct volume/total left ventricular volume) × 100%.

14. Olink proteome quantitative analysis

Myocardial tissue protein levels in the control, myocardial infarction (MI) model, and MI model with shock wave treatment groups were quantified using the Olink® Target 96 panel (Olink Proteomics; LC-Bio Technology Co., Ltd., Hangzhou, China) according to the manufacturer's protocol. Normalized Protein Expression (NPX) values, representing relative protein abundance in log2-scale arbitrary units, were generated with higher NPX values indicating greater protein abundance. Differentially expressed proteins (DEPs) were identified using the “OlinkAnalyze” R package. Differentially expressed proteins (DEPs) were initially analyzed using Welch's two-sample *t*-test. To control for multiple comparisons, *p*-values were adjusted using the Benjamini-Hochberg false discovery rate (FDR) method. Additionally, to screen candidate proteins for subsequent exploratory functional validation, an unadjusted nominal *p*-value < 0.05 was defined as an exploratory threshold. Following log2 transformation, hierarchical clustering analysis was performed on DEP expression profiles across samples. Heatmaps and volcano plots were generated using ggplot2 (v3.5.1). Gene ontology (GO) terms and Kyoto Encyclopedia of Genes and Genomes (KEGG) pathway were used to create gene sets. GO and KEGG protein functional enrichment analysis was performed on differentially expressed proteins, and the first 20 GO Terms with the smallest *p*-value were selected to draw bubble plots. PPI analysis was performed through the String database (threshold of 0.15), and the network was further analyzed using the MCODE plug-in (v2.0.3) and CytoHubba plug-in (v0.1) of Cytoscape (v3.10.3) software.

15. Statistical analysis

Sample size was determined by an *a priori* power analysis using G*Power software (version 3.1.9.7; Heinrich-Heine-Universität Düsseldorf, Germany). Based on our previous experimental experience and published evidence using analogous MI models, a large effect size (Cohen's *d* ≈ 1.8) was anticipated for the primary endpoint (myocardial infarct size). With an *α* level of 0.05 and a statistical power of 80% (1−*β* = 0.80), a minimum sample size of *n* = 6 per group was required to detect significant differences between the experimental groups.

GraphPad Prism software was used for statistical analysis, and the measurement data were expressed as mean ± standard deviation (Mean ± SD), and the degree of data dispersion was presented through the error line in the statistical graph. The methods of intergroup comparison were as follows: when comparing between two groups, independent samples *t*-test was applied to mutually independent samples, paired samples *t*-test was applied to two groups of samples before and after the treatment of the same batch of samples or paired design, and the premise of application was that the data approximately conformed to the normal distribution and had the chi-squaredness of variance, and Welch's corrected *t*-test was used for the unevenness of variance; the comparison of multi-groups firstly used one-way ANOVA to analyse the dispersion of a single independent data. One-way analysis of variance (ANOVA) was used to analyse whether there was a difference in the overall mean values between multiple levels of a single independent variable. Statistical differences were set at *p* < 0.05.

Furthermore, to minimize subjective bias, all quantitative analyses of histological sections, Western blots, and *in vitro* cellular assays were conducted by independent researchers blinded to the experimental group assignments.

## Results

1. *In vitro* experiments demonstrate that cardiac shock wave therapy activates CACs, upregulates angiogenic factor expression, and promotes angiogenesis

**Figure 1:**
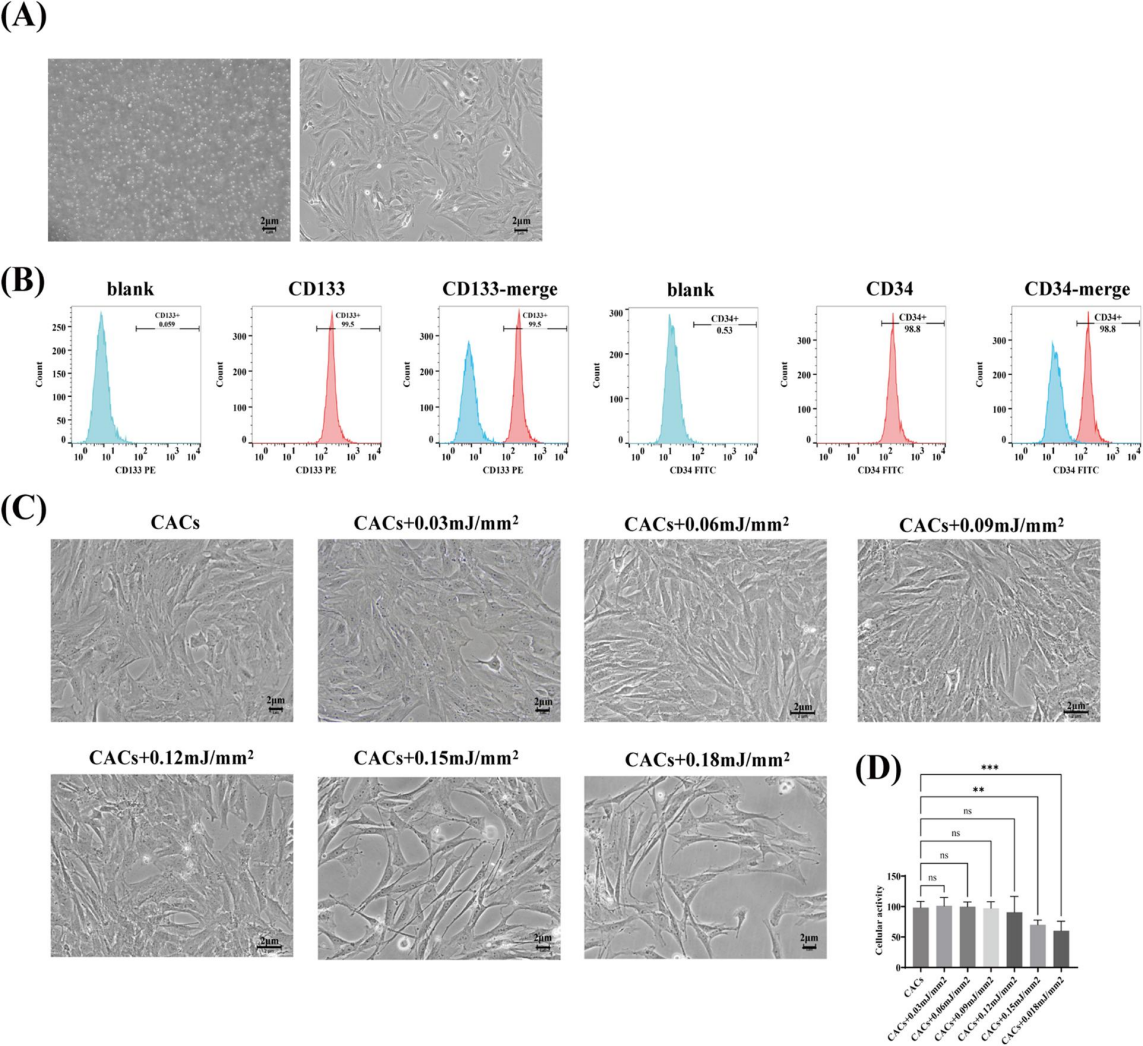
Validation of CACs isolation and optimization of shock wave parameters. **(A)** Isolatio n and culture of Coronary Angiogenic Cells (CACs) (detailed in method 2.2) Left diagram: The primary cells were round and suspended. Picture on the right: The adherent cells were passaged and amplified, showing a typical spindle-shaped and fibroblast-like morphology (rule*r* = 2 μm). **(B)** Flow cytometry was used to detect the expression of CACs surface markers CD133 and CD34, and Blank was an unstained blank control; CD133/CD34 was single staining group; CD133-merge/CD34-merge was the staining group, showing that the positive rate of CD133 was 91.6%, and the positive rate of CD34 was 96.8% (detailed in method 2.3). **(C)** Seismic wave treatment CACs with different energy flux densities were divided into control group (CACs), 0.03,0.06,0.09,0.12,0.15,0.18 mJ/mm^2^ seismic wave treatment groups (scale=2*μ*m) (detailed in method 2.4). **(D)** CCK-8 method was used to detect the cell proliferation activity of CACs after shock wave treatment (detailed in method 2.6). Statistical analysis was performed using *t*-test or one-way analysis of variance combined with Tukey *post hoc* test. Data were expressed as Mean ± SD, n = 3. ns, *p* > 0.05; *p < 0.05; **p < 0.01; ***p < 0.001; ****p < 0.0001.

To investigate the effects of cardiac shock wave therapy (CSWT) on CACs function, CACs were isolated from healthy rat bone marrow and expanded *in vitro* (Figure 1A). Freshly isolated CACs appeared as circular suspended cells, which gradually adhered, elongated, and adopted a spindle-like or irregular morphology upon culture. To confirm CACs purity, expression of the characteristic surface markers CD34 and CD133 was assessed by flow cytometry (Figure 1B). Following a stringent gating strategy (Supplementary Figure S1), the results indicated that over 99% of the isolated cells were double-positive for both CD34 and CD133. To optimize the shock wave energy parameters, we performed a dose-response analysis using the CCK-8 assay to evaluate the effect of various energy levels on CACs viability (Figures 1C,D). No significant cytotoxic effects or changes in proliferation were observed at densities from 0.03 to 0.12 mJ/mm^2^ (*p* > 0.05), indicating a safe therapeutic window. However, higher energy levels proved detrimental, with proliferation decreasing by 22.31% at 0.15 mJ/mm^2^ (*p* < 0.01) and by 33.4% at 0.18 mJ/mm^2^ (*p* < 0.001). Therefore, 0.09 mJ/mm^2^ was determined as the optimal parameter for subsequent *in vitro* experiments (1 Hz). This dose lies within a safe non-cytotoxic range and, consistent with prior reports (17), elicits robust functional activation of progenitor cells without triggering apoptosis.

**Figure 2:**
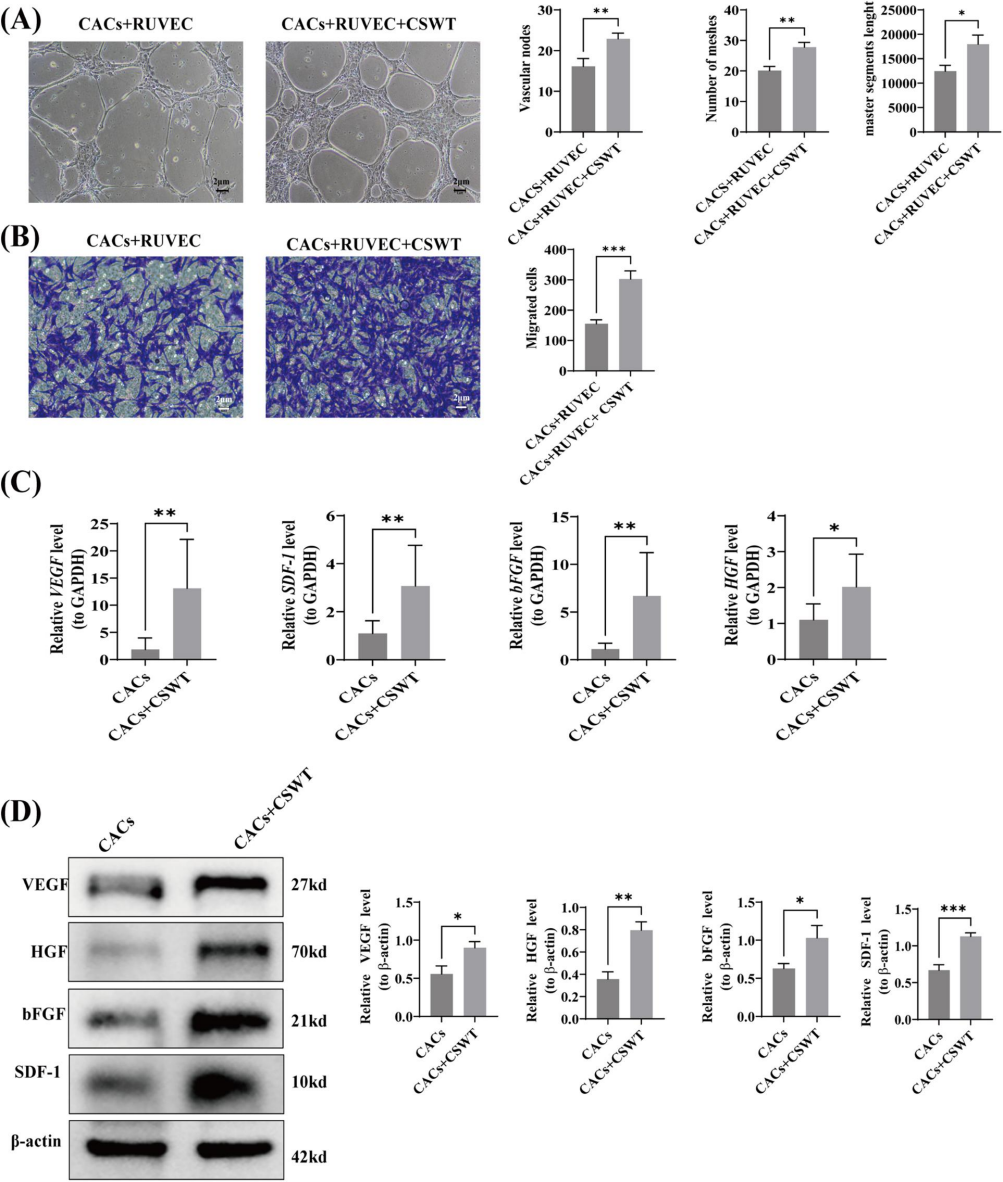
*In vitro* validation of CSWT enhancing the pro-angiogenic function of CACs via upregulation of pro-angiogenic factor expression. **(A)** Tube formation assay was used to detect the effect of CSWT on the angiogenesis ability of CACs and RUVECs co-culture system. Left: control CACs and RUVEC co-culture (CACs + RUVEC); right: CSWT treated CACs and RUVEC co-culture (CACs + RUVEC + CSWT). The number of vascular nodes (Vascular nodes), the number of vascular networks (Number of meshes) and the length of main branches (master segments length) were counted in the histogram (scale: 2μm) (method 2.8 details). **(B)** Transwell migration assay was used to detect the effect of CSWT on the migration ability of CACs cells in the co-culture system. Left: control group; right: CSWT treatment group, DAPI staining showed the nucleus (blue) (method 2.7 details) (scale: 2μm). **(C)** RT-qPCR was used to detect the mRNA expression levels of angiogenesis-related factors VEGF, SDF-1, bFGF and HGF in CACs after CSWT treatment, with GAPDH as an internal reference (method 2.12 details). **(D)** Western blot was used to detect the protein expression levels of VEGF, HGF, bFGF and SDF-1 in CACs after CSWT treatment, with *β*-actin as an internal reference (method 2.11 details). *t*-test or one-way analysis of variance combined with Tukey *post hoc* test was used for statistical analysis. The data were expressed as Mean ± SD, n = 3. ns, *p* > 0.05; *p < 0.05; **p < 0.01; ***p < 0.001; ****p < 0.0001.

To explore the role of shock waves in regulating angiogenesis, shock wave-pretreated CACs were co-cultured with rat umbilical vein endothelial cells (RUVECs) to evaluate their impact on endothelial cell vascularization capacity. The angiogenic potential of RUVECs in the co-culture system was assessed using an *in vitro* tube formation assay. Since the Transwell system (0.4 μm pore size) prevents direct cell-cell contact, the results (Figure 2A) suggested a soluble factor-mediated paracrine effect, where CACs pretreated with 0.09 mJ/mm^2^ shock waves significantly promoted the formation of more extensive vascular networks in RUVECs. Compared to the control group, the shock wave intervention group exhibited a significant increase in the number of vascular branches (control: 21.33 ± 1.10; shock wave: 29 ± 1.29; *p* < 0.01), trunk length (control: 12,441.41 ± 997.61; shock wave: 17,960.55 ± 1,539.02; *p* < 0.01), and nodes (control: 16.11 ± 1.59;: 22.89 ± 1.13; *p* < 0.01). To evaluate the effect of shock wave treatment on CAC migratory ability, a Transwell migration assay was performed (Figure 2B). The number of migrated CACs was significantly higher in the shock wave-stimulated group (302 ± 17.72) than in the control group (155 ± 30.48; *p* < 0.05), indicating that shock wave stimulation markedly enhanced CACs migration. For mechanistic insights, qPCR analysis revealed that shock wave treatment significantly upregulated the mRNA expression of angiogenesis-related factors (VEGF, HGF, bFGF, and SDF-1) in CACs (Figure 2C; *p* < 0.01). Western blot analysis further confirmed that CSWT promoted the mRNA and protein expression of VEGF, HGF, bFGF, and SDF-1 in CACs (Figure 2D; full-length uncropped blots are presented in Supplementary Figure S2; *p* < 0.01). These *in vitro* findings provide foundational support for the *in vivo* mechanisms.

2. *In vivo* validation of extracorporeal cardiac shock wave therapy in reducing infarct size and promoting structural preservation following acute myocardial infarction

**Figure 3:**
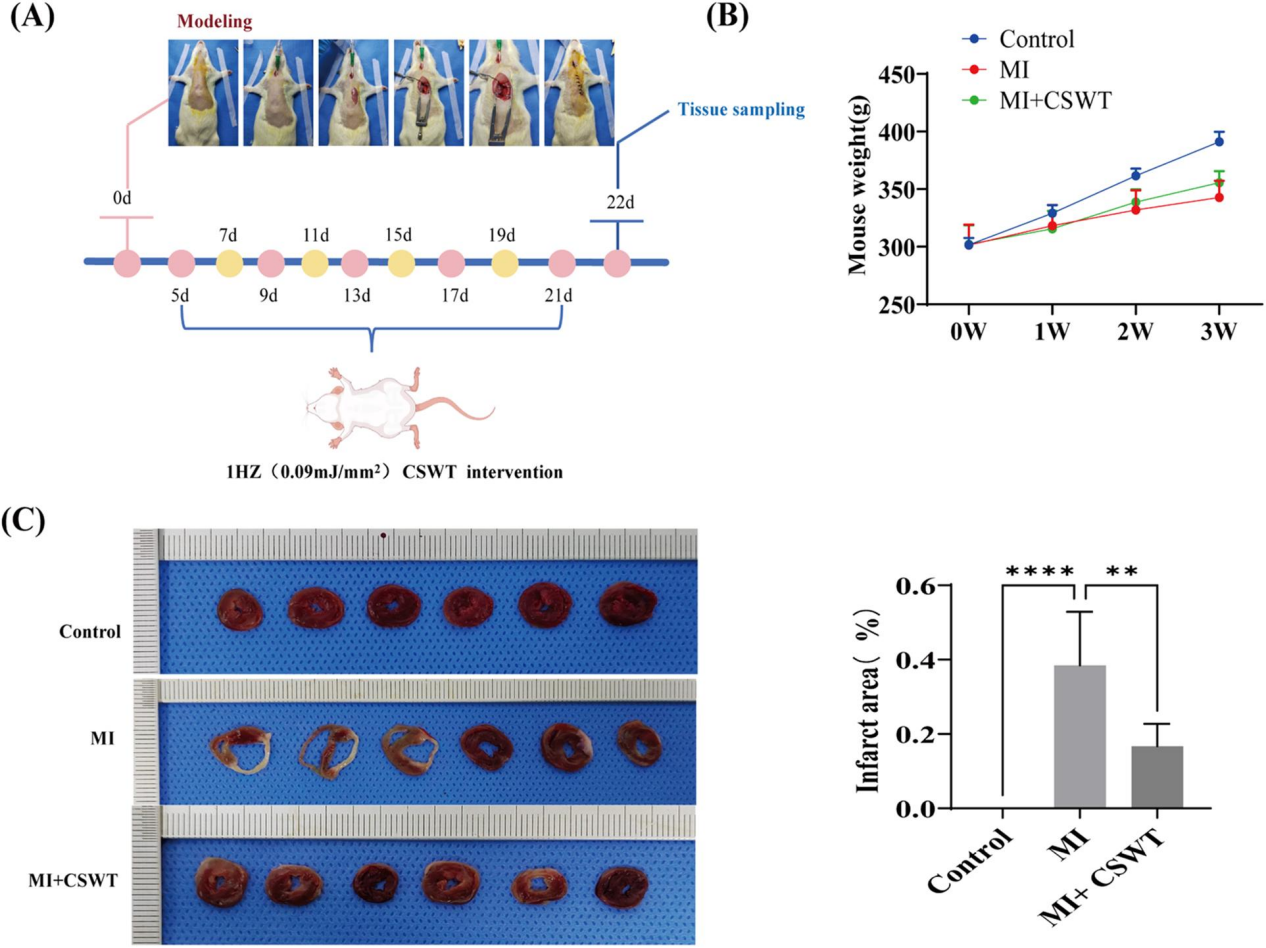
Extracorporeal cardiac shock wave therapy remodels post-infarction myocardium in rats. **(A)** The overall process and intervention plan of animal experiments. The above is a schematic diagram of the surgical steps constructed for the myocardial infarction model; the following was the experimental time axis: MI model was constructed at 0d, and CSWT intervention was performed with parameters of 1 Hz and 0.09 mJ/mm^2^ from the 5th day after operation, once every 4 days until the 21st day; samples were taken on the 22 nd day (see method 2.1 for details). **(B)** The weight change curve of mice in each group during the experiment was recorded in weeks (W). **(C)** TTC staining results and quantitative analysis of infarct size in myocardial tissue of mice in each group. The upper part was the normal control group, the middle part was the MI model group, and the lower part was the MI + CSWT intervention group. The histogram showed the percentage of myocardial infarction area (%) in the three groups, and CSWT intervention could significantly reduce the myocardial infarction area (see method 2.13). Statistical analysis was performed using *t*-test or one-way analysis of variance combined with Tukey *post hoc* test. The data were expressed as Mean ± SD. Control was the normal control group, MI was the myocardial infarction model group, and MI + CSWT was the shock wave intervention group, n = 6. ns, *p* > 0.05; *p < 0.05; **p < 0.01; ***p < 0.001; ****p < 0.0001.

Building on the *in vitro* activation of CACs, we further validated the *in vivo* modulation of myocardial function by shock wave therapy. A rat model of acute myocardial infarction (MI) was established (Figure 3A). Post-modeling, shock wave interventions were administered, and body weights were monitored over time (Figure 3B). Although body weights in both the MI and MI + CSWT groups exhibited a downward trend compared to the control group, these differences did not achieve statistical significance. TTC staining (Figure 3C) revealed a significantly larger infarct size in the MI group (38.5% ± 12.45%) compared to the control group (0%; *p* < 0.001). In contrast, the MI + CSWT group displayed a markedly reduced infarct size (16.75% ± 5.66%) relative to the MI group (*p* < 0.01).

**Figure 4:**
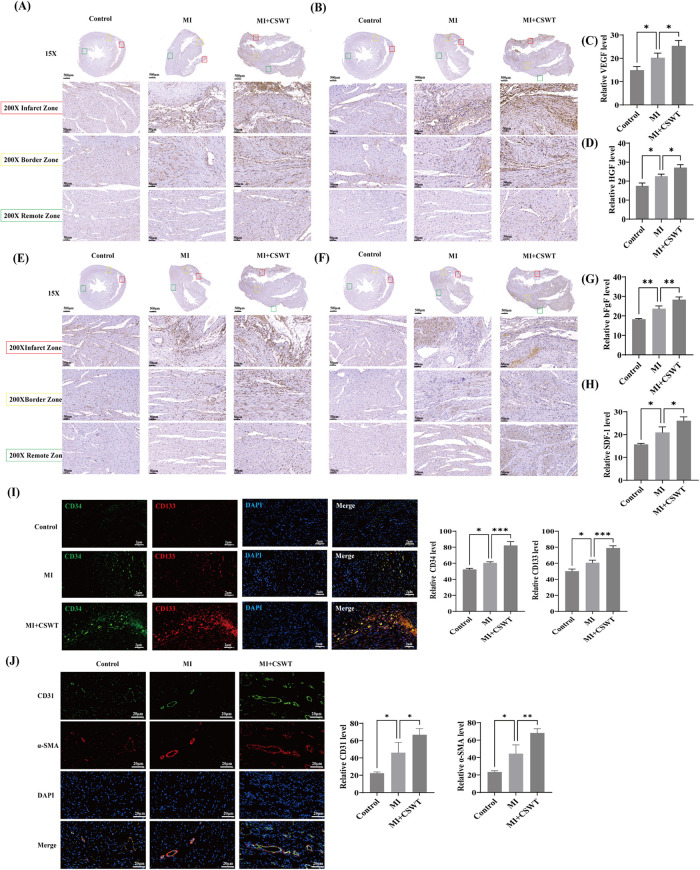
Extracorporeal cardiac shock wave therapy modulates CACs activation and promotes neovascularization. **(A,B)** Immunohistochemical staining was performed to detect the expression levels of VEGF **(A)** and HGF **(B)** in rat myocardial tissues of each group. The 15× magnification shows the overall view of myocardial tissue (Scale ba*r* = 500 μm), and the 200× magnification presents the positive expression distribution in the infarcted area (red box), border zone (yellow box), and remote area (green box), respectively (Scale ba*r* = 50 μm) (detailed in Method 2.9). **(C,D)** Semi-quantitative analysis results of VEGF **(C)** and HGF **(D)** protein expression levels (detailed in Method 2.15). **(E,F)** Immunohistochemical staining was used to detect the expression levels of bFGF **(E)** and SDF-1 **(F)** in rat myocardial tissues of each group. The 15× magnification shows the overall view of myocardial tissue (Scale ba*r* = 500 μm), and the 200× magnification presents the positive expression distribution in the infarcted area (red box), border zone (yellow box), and remote area (green box), respectively (Scale ba*r* = 50 μm) (detailed in Method 2.9). **(G,H)** Semi-quantitative analysis results of bFGF **(G)** and SDF-1 **(H)** protein expression levels (detailed in Method 2.15). **(I)** Immunofluorescence staining was used to detect the co-expression of CD34 (green) and CD133 (red) in myocardial tissues of each group, with DAPI (blue) staining the cell nuclei. The bar graph on the right shows the relative quantitative results of CD34⁺/CD133⁺ cells (Scale ba*r* = 2 μm) (detailed in Method 2.10). **(J)** Immunofluorescence staining was used to detect the expression of CD31 (green) and *α*-SMA (red) in myocardial tissues of each group, with DAPI (blue) staining the cell nuclei. The bar graph on the right shows the relative quantitative results of CD31⁺ and *α*-SMA⁺ cells (Scale ba*r* = 20 μm) (detailed in Method 2.10). Statistical analysis was performed using *t*-test or one-way analysis of variance combined with Tukey *post hoc* test. Data were expressed as Mean ± SD, *n* = 3。ns, *p* > 0.05; *p < 0.05; **p < 0.01; ***p < 0.001; ****p < 0.0001.

To further elucidate the regulatory mechanisms of extracorporeal cardiac shock wave therapy on CACs chemotaxis and activation, immunohistochemical (IHC) staining and immunofluorescence (IF) techniques were employed to systematically assess changes in angiogenesis-related factors and CACs-specific markers. IHC results demonstrated elevated expression levels of VEGF, HGF, bFGF, and SDF-1 in the MI model group compared to the control group (Figures 4A–H; *p* < 0.01), with further increases in these factors following CSWT treatment relative to the MI group (Figures 4A–H; *p* < 0.01). Additionally, IF results showed that expression levels of the CACs markers CD34 and CD133 in the MI + CSWT group were significantly higher than in the control and MI groups (Figure 4I; *p* < 0.01). IF staining for CD31 and *α*-SMA co-localization indicated that expression levels of both CD31 and *α*-SMA in the MI + CSWT group were markedly elevated compared to the control and MI groups (Figure 4J; *p* < 0.05). Moreover, CD31/*α*-SMA co-localization was also significantly increased relative to the other two groups (Figure 4J; *p* < 0.05).

3. Proteomic profiling of extracorporeal cardiac shock wave therapy effects on CACs function using Olink technology.

**Figure 5:**
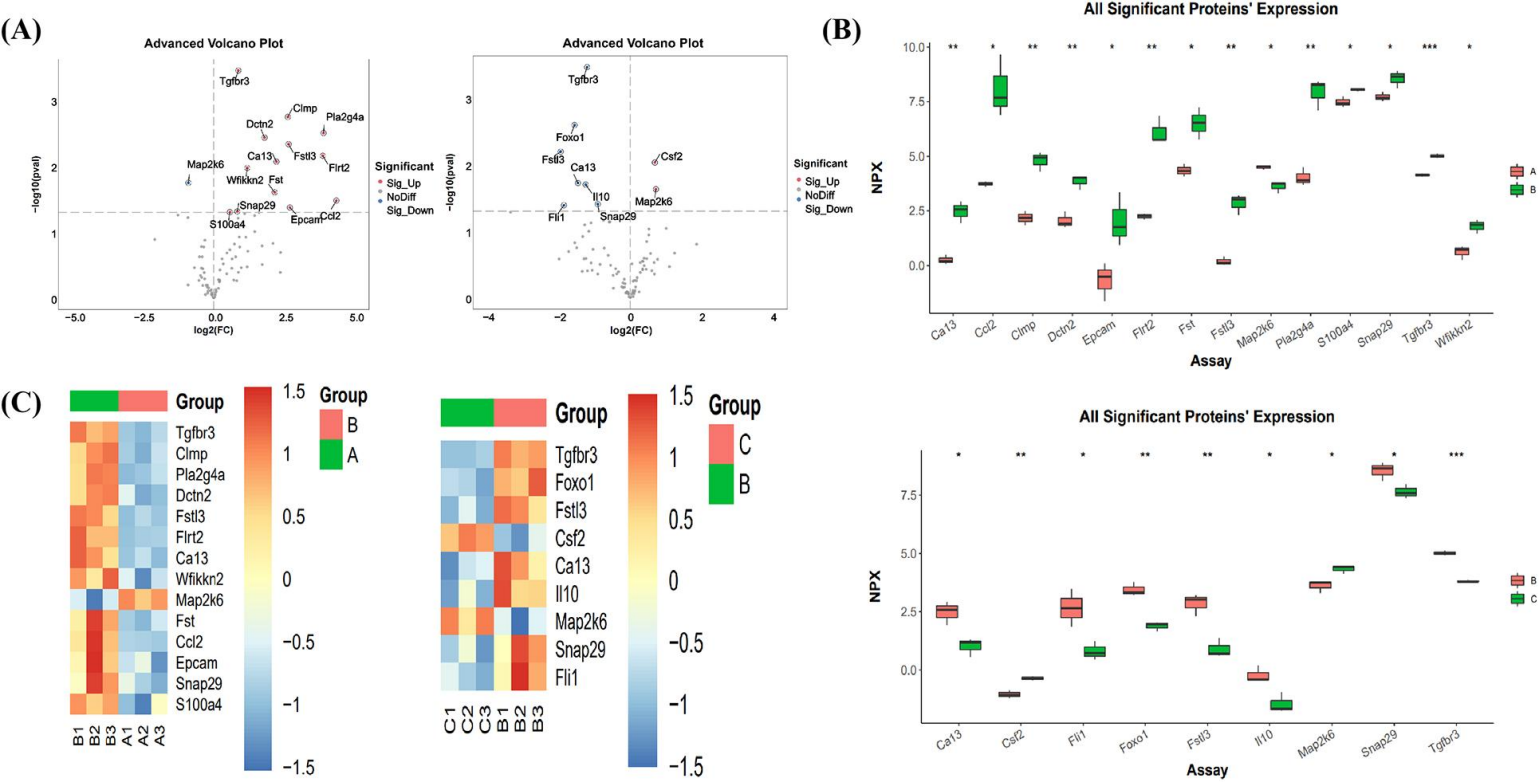
**(A)** volcano plot showed the distribution of differentially expressed proteins between the two groups. The abscissa is the difference multiple (log₂FC), and the ordinate was the significant level (-log₁₀P). Red dots represent significantly up-regulated proteins, blue dots represent significantly down-regulated proteins, and gray dots are proteins with no statistical difference. **(B)** Boxplot shows the expression levels of all significantly different proteins in each group. The upper figure: group A (control group) VS group B (model group), the lower figure: group B (model group) VS group C (CSWT group). The ordinate is the protein quantitative signal value (NPX), and the abscissa is the name of each protein, showing the difference in expression between groups and statistically significant markers. **(C)** Heatmap cluster analysis of differentially expressed proteins, left figure: group A (control group) VS group B (model group), right figure: group B (model group) VS group C (CSWT group). The color gradient represents the protein expression level (red for high expression, blue for low expression), each row represents a protein, and each column represents a sample, clearly showing the inter-group differences and clustering patterns of protein expression.

To elucidate the molecular mechanisms underlying shock wave therapy, we performed Olink proteomic analysis to further investigate the regulatory effects of *in vitro* cardiac shock wave treatment on CACs function. Initially, following multiple comparison correction via the Benjamini-Hochberg method, only a single protein (TGFBR3) achieved strict statistical significance at an FDR < 0.05 (Supplementary Table S1), likely due to the limited sample size and inherent biological variation. Because relying solely on this single node would preclude meaningful network and pathway enrichment analyses, we utilized our predefined exploratory threshold (unadjusted nominal *p*-value < 0.05) to capture a broader spectrum of potentially relevant biological targets. Consequently, utilizing our predefined exploratory threshold (unadjusted nominal *p*-value < 0.05), differential expression analysis (Figures 5A,B) identified 14 differentially expressed proteins in the model group (B) compared to the control group (A), of which 13 were upregulated and only Map2k6 (*P* = 0.0178) was downregulated. Applying these same exploratory criteria, in the CSWT group (C) compared to the model group (B), 9 differentially expressed proteins were screened, with 7 downregulated, whereas CSF2 and Map2k6 were upregulated. The differential protein heatmap further demonstrated that expression levels of Map2k6 (*P* = 0.0235) and CSF2 (*P* = 0.0093) were significantly higher in the CSWT group (C) than in the model group (B) (Figure 5C; *p* < 0.01).

4. Identification of key proteins regulating CACs activation for post-myocardial infarction neovascularization through proteomic screening.

**Figure 6:**
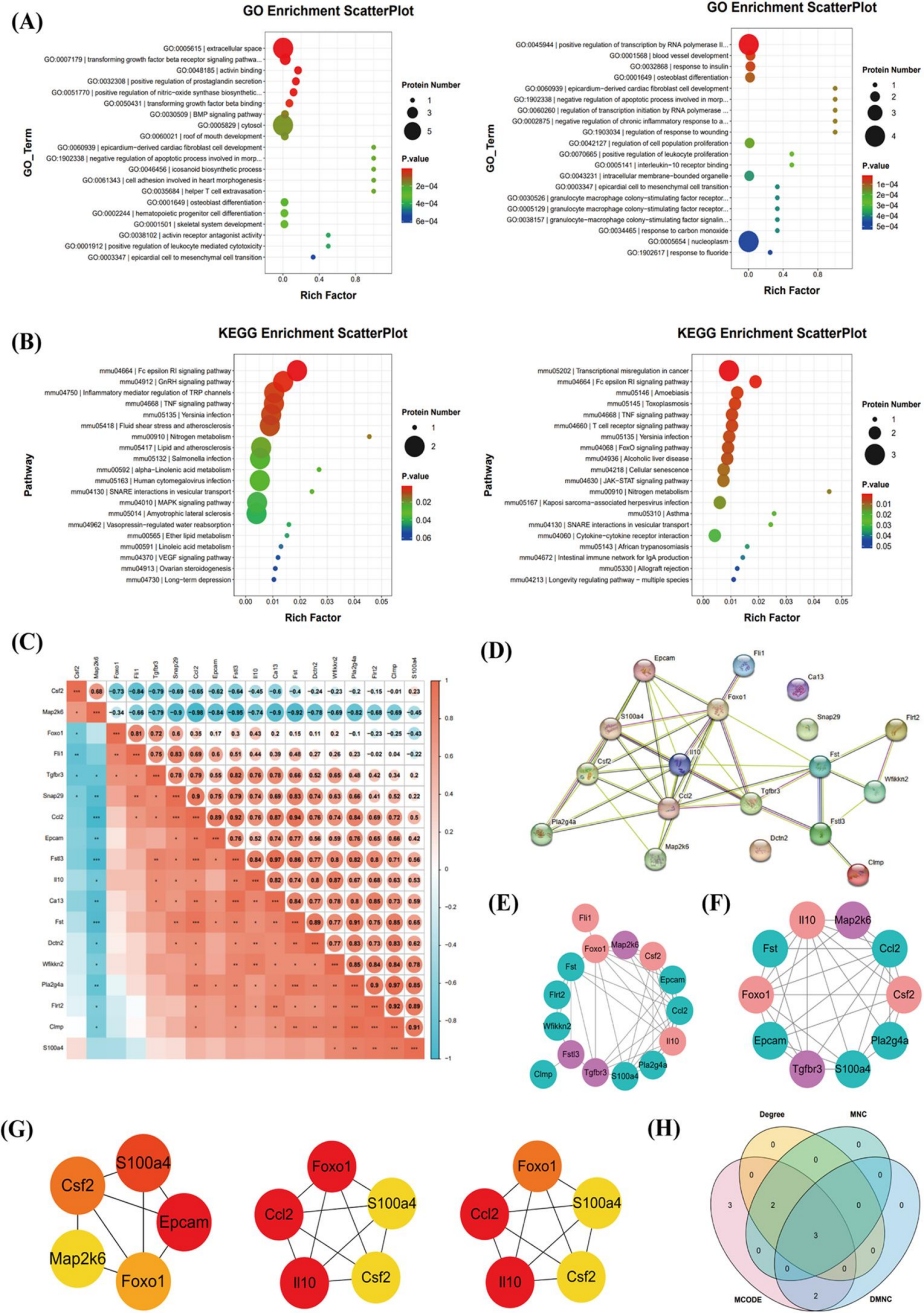
Olink proteomics analysis reveals key molecules regulating CAC function following *in vitro* cardiac shock wave treatment. **(A)** GO function enrichment bubble diagram of differentially expressed proteins. The abscissa is the enrichment factor (Rich Factor, representing the degree of enrichment), and the ordinate is the GO entry (including biological processes, cell components, and molecular functions); the bubble size represented the number of proteins enriched to this item, and the color represented the significant *P*-value). **(B)** KEGG pathway enrichment bubble diagram of differentially expressed proteins. The abscissa is the enrichment factor, and the ordinate is the name of the KEGG signaling pathway; the bubble size represented the number of proteins enriched in the pathway, and the color represented the significant *P*-value. **(C)** Pearson correlation analysis heat map between differentially expressed proteins. The color gradient represents the correlation coefficient of protein expression (red is positively correlated, blue is negatively correlated, and the circle size and color depth reflect the degree of correlation. **(D)** Protein-protein interaction (PPI) network of differentially expressed proteins based on STRING database. The node represents differentially expressed proteins, the connection represents the interaction between proteins, and the thickness of the connection corresponds to the confidence level of the interaction. **(E)** Visualization results of PPI network by Degree algorithm of Cytoscape software, in which green, red and purple nodes represent differentially expressed proteins in different comparison groups. **(F,G)** Cytoscape software was used to screen core protein interaction modules by various algorithms (MCC, DMNC, MCODE). The node color represents the importance of the protein in the network (Degree value), and the connection represents the interaction between the proteins. **(H)** Four algorithms including Degree, MCC, DMNC and MCODE were used to screen the Venn diagram of key regulatory proteins. Different colors represent different algorithms, and the overlapping part is the core regulation of common recognition of all algorithms.

To further identify key molecular markers involved in neovascularization, functional enrichment and network analyses were conducted based on the differential expression results from Olink proteomics. GO enrichment analysis (Figure 6A) revealed that differentially expressed proteins between the model group (B) and control group (A) were significantly enriched in angiogenesis-related biological processes, such as “epicardial cell to mesenchymal cell transition” and “vascular endothelial growth factor-mediated vascular development,” indicating activation of neovascularization pathways during acute infarction. KEGG pathway enrichment analysis (Figure 6B) showed that differential proteins between the model group (B) and control group (A) were enriched in 62 signaling pathways, including the Fc epsilon RI signaling pathway, GnRH signaling pathway, and MAPK pathway; differential proteins between the CSWT group (C) and model group (B) were enriched in 68 pathways, primarily related to the TNF signaling pathway and T cell receptor signaling pathway. Further intersection analysis of differential proteins from the two comparison groups identified 18 key proteins potentially involved in regulating CACs activation (Figure 6C). Correlation analysis demonstrated that CSF2 exhibited significant positive correlations with Map2k6 (r = 0.68) and S100A4 (r = 0.23), but negative correlations with all other proteins. Construction of the protein-protein interaction (PPI) network (Figure 6D) revealed close interactions among the differential proteins. Cytoscape was used to visualize the PPI network (Figure 6E), and based on multiple network topology algorithms, three core proteins—S100A4, CSF2, and FOXO1—were identified (Figures 6F–H).

**Figure 7:**
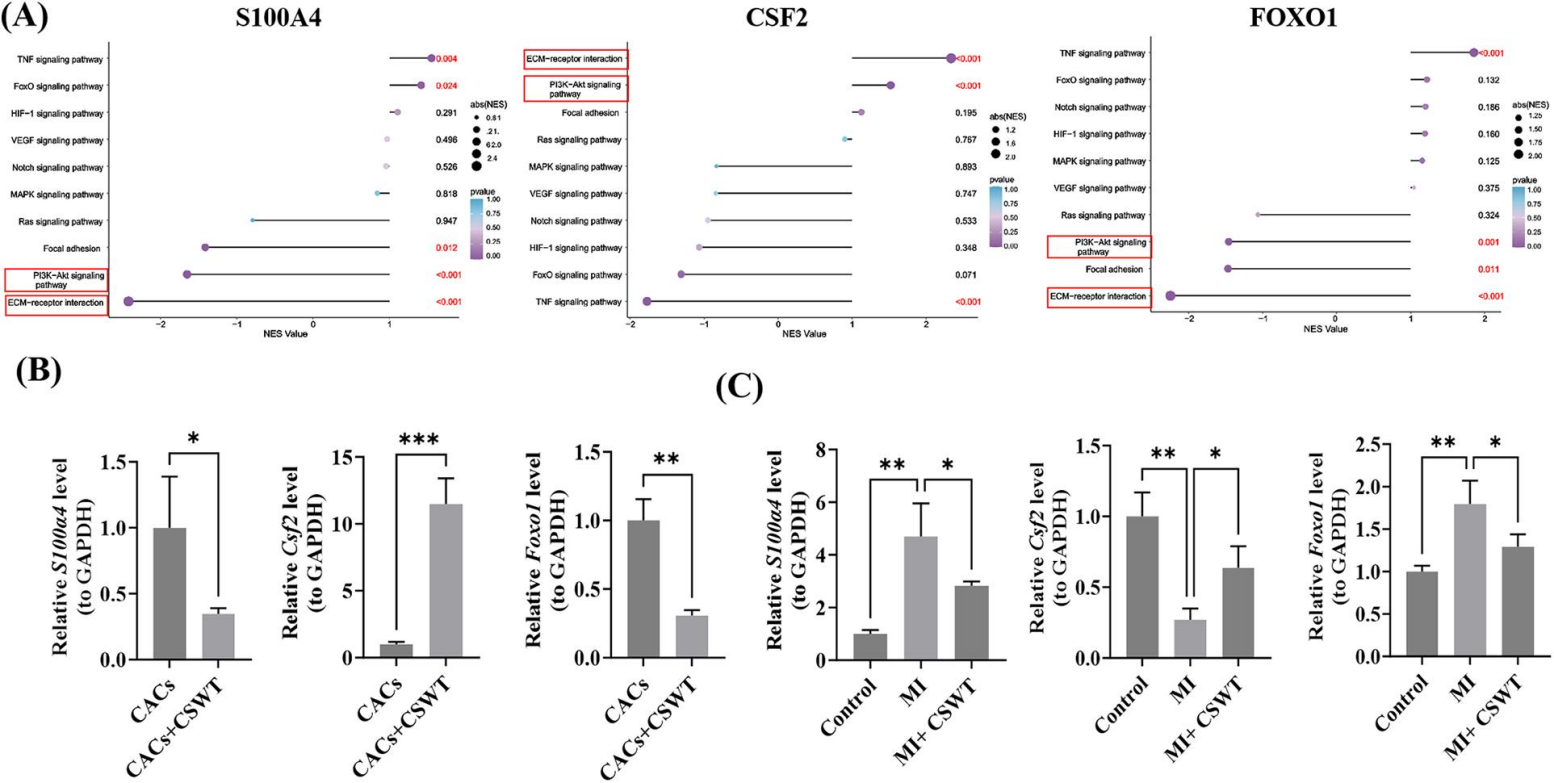
qPCR validation of key factor expression (as described in methods 2.12). **(A)** Gene set enrichment analysis (GSEA) of key target proteins. The enrichment intensity (NES value) of S100A4, CSF2 and FOXO1 in different signaling pathways was shown respectively, and the color gradient represented the enrichment significance (*P*-value). The results showed that the above molecules were significantly enriched in the PI3K-Akt signaling pathway and ECM-receptor interaction pathway. **(B)** RT-qPCR as used to detect the mRNA expression levels of CSF2, S100A4 and F0XO1 in CACs before and after CSWT treatment, and GAPDH was used as an internal reference. **(C)** *in vivo* animal level verification: RT-qPCR was used to detect the mRNA expression levels of CSF2, S100A4 and FOXO1 in myocardial tissue of Control group, MI model group and MI + CSWT intervention group, with GAPDH as internal reference. *t*-test or one-way analysis of variance combined with Tukey *post hoc* test was used for statistical analysis. The data were expressed as Mean ± SD, n = 3. ns, *p* > 0.05; *p < 0.05; **p < 0.01; ***p < 0.001; ****p < 0.0001.

**Figure 8:**
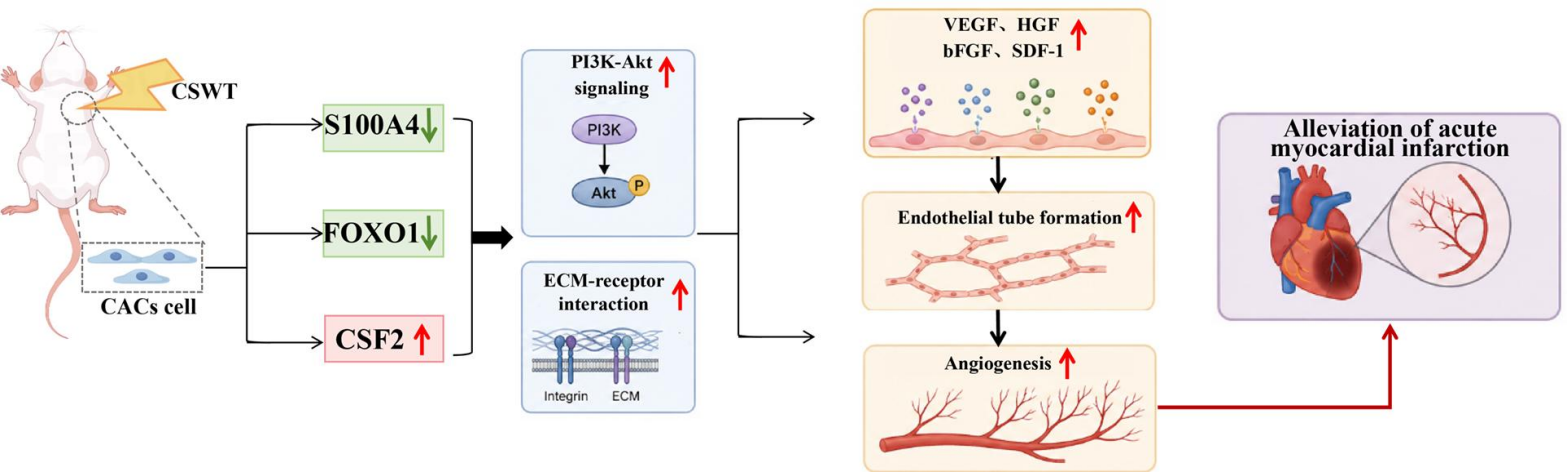
CSWT promotes angiogenesis in CACs by modulating the S100A4/FOXO1/CSF2 network and its associated PI3K-Akt and ECM-receptor interaction pathways.

To further explore their potential regulatory mechanisms in angiogenesis, single-gene set enrichment analysis (GSEA) was performed. The results (Figure 7A) revealed that S100A4, CSF2, and FOXO1 were all significantly enriched in the ECM-receptor interaction pathway and the canonical pro-survival/pro-angiogenic PI3K-Akt signaling pathway. Notably, S100A4 (*P* < 0.001 for both) and FOXO1 (*P* = 0.001 and *P* < 0.001, respectively) exhibited significant negative correlations with both the PI3K-Akt and ECM-receptor interaction pathways. Similarly, CSF2 (*P* < 0.001 for both) also displayed negative correlations with these two signaling cascades. Subsequently, to validate the regulatory effects of shock wave intervention on these key genes, qPCR assays were performed at both cellular and animal levels. In CACs, shock wave treatment significantly upregulated *Csf2* mRNA expression (*P* = 0.0007), while downregulating *S100a4* (*P* < 0.05) and *Foxo1* (*P* < 0.01) (Figure 7B). Consistent with these *in vitro* findings, shock wave intervention in the acute MI rat model led to the upregulation of *Csf2* (*P* = 0.0349) and the downregulation of *S100a4* (*P* = 0.0425) and *Foxo1* (*P* = 0.0050) (Figure 8). Proposed mechanism illustrated by the graphical abstract: In Circulating Angiogenic Cells (CACs), elevated expression of S100A4 and FOXO1 may inhibit the PI3K-Akt signaling and extracellular matrix (ECM)-receptor interaction pathways, thereby reducing the secretion of angiogenesis-related factors. Similarly, decreased expression of CSF2 may also suppress these two pathways, leading to a reduction in angiogenic factor release. Cardiac shock wave therapy (CSWT) may reverse this impaired state by upregulating CSF2 and targetedly downregulating S100A4 and FOXO1. This orchestrated modulation facilitates the secretion of pro-angiogenic factors and enhances the tube formation capacity of CACs, ultimately promoting neovascularization and attenuating structural damage in acute myocardial infarction (AMI).

## Discussion

In this study, we present findings exploring the hypothesis that CSWT modulates post-infarction neovascularization not merely through isolated signaling events, but by orchestrating a systems-level shift in the myocardial microenvironment. Our investigation integrates high-throughput proteomics (Figure 5) with functional cellular assays (Figure 1) to suggest a coordinated repair mechanism: CSWT specifically modulates the S100A4/CSF2/FOXO1 protein network within the ischemic tissue (Figure 6), which in turn optimizes the recruitment and paracrine functionality of circulating angiogenic cells (CACs) (Figure 4). These findings extend prior mechanistic insights by mapping the specific molecular “soil” that fosters an endogenous, pro-angiogenic repair response in the setting of acute myocardial infarction (AMI).

Evaluating the effects of CSWT within a preclinical context, our *in vitro* experiments, optimized-dose CSWT significantly enhanced CACs migration and tube formation capacity (Figure 1). Notably, while CSWT facilitated these functional improvements, it did not significantly alter cell proliferation under our current non-stressed experimental setup (Figures 1C,D). This selective activation suggests that in a normoxic environment, CSWT preferentially modulates factors such as VEGF, HGF, and bFGF to drive recruitment and network formation rather than direct cell-cycle expansion (13, 15). Indeed, EPC cell-cycle kinetics are exquisitely sensitive to local oxygen tension. The HIF-1*α*–TWIST–p21 signaling axis functions as a critical checkpoint in this context (20). Under standard normoxic conditions, EPCs undergo replicative senescence driven by p21 accumulation, which arrests cell-cycle progression. Conversely, a hypoxic microenvironment—mimicking the physiological bone marrow niche—triggers HIF-1*α*-dependent TWIST expression. This induction directly represses p21 and upregulates key cell-cycle drivers, including Cyclin D1 and Cyclin E, thereby facilitating S-phase entry and fueling EPC hyperproliferation (20). This discrepancy highlights the potential condition-specific nature of shock wave therapy, where mechanical stimulation may require synergy with ischemic stress signals to trigger hyperproliferation.

Translating these cellular insights to the *in vivo* setting, our Transwell and expression data provide associative support for the paracrine hypothesis, although the specific causal secretory factors remain to be definitively isolated. In the rat AMI model, CSWT induced a synchronized upregulation of core angiogenic drivers, including VEGF and SDF-1 (Figure 2). As described by (12), these factors serve as core drivers of the angiogenic cascade. Crucially, consistent with (1), our findings support the consensus that bone-derived stem cells facilitate repair primarily via paracrine signaling rather than direct transdifferentiation into cardiovascular lineages. Structurally, this molecular shift translated into a profound therapeutic benefit, reducing infarct size from approximately 38.5% to 16.75% (Figure 3). This preservation was accompanied by a marked enrichment of CD34+/CD133 + progenitor cells and CD31+/*α*-SMA + mature vessels in the peri-infarct zone (Figure 4), suggesting that the therapy effectively recruits CACs to the ischemic niche to fuel neovascularization.

To further provide mechanistic insights into this repair response, our Olink proteomic profiling identified a distinct signature of 18 differentially expressed proteins (Figure 5). Network topology analysis via Cytoscape subsequently pinpointed S100A4, CSF2, and FOXO1 as central signaling hubs (Figure 6). Validation via qPCR indicated a consistent expression pattern, suggesting its potential role as a regulatory node for repair (Figure 7, Figure 8). Specifically, the marked upregulation of CSF2 (GM-CSF) likely facilitates the mobilization of bone marrow-derived stem cells and enhances collateral circulation formation (18). CSF2 acts as a critical bridge between systemic immune mobilization and local repair, potentially mediating the transition from the inflammatory phase to the proliferative phase of healing. However, we acknowledge that the dual effects of GM-CSF warrant caution, as excessive expression could exacerbate inflammation (21), underscoring the importance of the regulated upregulation observed in our study.

Moreover, the modulation of S100A4 and FOXO1 suggests that CSWT fundamentally reprograms the ischemic microenvironment. Although S100A4 promotes early cardiomyocyte survival (22), its persistent expression drives fibroblast activation and fibrosis. Our finding that CSWT downregulates S100A4 (Figure 7) suggests it has the potential to attenuate excessive fibrosis and mitigate subsequent pathological remodeling (23). Alongside this anti-fibrotic effect, FOXO1 acts as an intrinsic negative checkpoint for angiogenesis (24). The concomitant downregulation of FOXO1 and elevation of angiogenic factors (Figure 2, Figure 7) indicates that CSWT effectively removes this angiogenic brake.By integrating these individual findings into a unified S100A4/CSF2/FOXO1 axis, we offer an exploratory perspective on how CSWT may modulate the myocardial microenvironment beyond canonical PI3 K/Akt signaling (17). Furthermore, our proteomic analysis indicates the potential involvement of broader signaling networks, with enrichment in the MAPK and TNF pathways (Figure 5); however, their precise functional contributions and causal links to CSWT-mediated repair warrant further investigation. This systems-level modulation may offer a preliminary conceptual framework for multi-target therapies, potentially complementing traditional pharmacological approaches that focus on isolated receptors. Such a network-based perspective provides a starting point for the future development and validation of integrated therapeutic strategies in cardiac repair.

Grounded in our findings and extant clinical literature, CSWT emerges as a non-invasive adjunctive therapy with substantial potential. While current clinical applications (e.g., the CAST-HF trial) predominantly target chronic ischemic heart disease (21), our study suggests that CSWT may hold significant therapeutic promise in the acute phase (AMI). A critical challenge in current AMI management is the “no-reflow” phenomenon, often attributed to microvascular obstruction and rarefaction despite patent epicardial arteries (22). By promoting the regeneration and maturation of the microvasculature—as evidenced by our observed increase in CD31 + capillary density and *α*-SMA + vascular stabilization (Figure 4)—CSWT targets the structural root of microvascular dysfunction (23). This suggests that CSWT could serve as a complementary mechanism to mechanical revascularization strategies, such as percutaneous coronary intervention (PCI), potentially improving tissue perfusion at the microcirculatory level. Furthermore, the key proteins identified herein (e.g., S100A4 monitoring) hold promise as potential biomarkers for tailoring therapeutic regimens in the era of precision medicine (25).

From a translational perspective, our findings provide a rationale to optimize cell-based therapies. Although major clinical trials (e.g., TOPCARE-AMI, BOOST) demonstrate the safety and feasibility of autologous bone marrow mononuclear cell (BMMNC) infusion for myocardial infarction, poor cell homing often limits their overall efficacy. Because CSWT preconditioning enhances the recruitment of infused human progenitor cells via local chemokine upregulation (26), we propose a dual-priming strategy to synergize these modalities. Localized CSWT could help prime the human cardiac niche prior to cell infusion by establishing a robust chemotactic gradient of SDF-1, VEGF, and endogenous CSF2. Concurrently, *ex vivo* hypoxic preconditioning might reverse replicative senescence in patient-derived BMMNCs via the HIF-1*α*-TWIST-p21 axis (20). Coupling this with CSF2 stimulation could further sustain a proliferative phenotype through PI3 K/MAPK signaling (27). Ultimately, integrating CSWT-mediated niche priming with *ex vivo* cell empowerment holds promise to mitigate current homing barriers, synergistically driving targeted neovascularization and improving clinical outcomes.

Notwithstanding the multifaceted regulatory potential of CSWT in MI repair unveiled herein, several limitations merit acknowledgment. Foremost, regarding mechanistic validation, while our current findings indicate a strong association between CSWT and the S100A4/CSF2/FOXO1 network, the absence of direct targeted interventions (e.g., FOXO1 knockdown, S100A4 silencing, or CSF2 neutralization), conditioned media transfer experiments, and comprehensive secretome profiling precluded the establishment of definitive causality. Second, concerning cell characterization, our identification of CACs primarily relied on CD34/CD133 expression and functional assays; while this selectively enriches for primitive progenitors, the lack of multi-color flow cytometry panels (e.g., incorporating VEGFR2, CD31, and CD45) means we cannot provide a more granular definition of specific sub-populations within this inherently heterogeneous, hematopoietic-derived progenitor pool. Third, regarding *in vivo* outcomes, our assessment focused primarily on acute structural endpoints (infarct size via TTC staining) and molecular markers. The current study lacks comprehensive *in vivo* cardiac functional evaluations, such as longitudinal echocardiography (assessing Ejection Fraction or Fractional Shortening) and hemodynamic monitoring. Fourth, regarding theproteomic profiling, limited sample size and inherent biological variability resulted in only a single differentially expressed protein (TGFBR3) reaching statistical significance after False Discovery Rate (FDR) correction. Because relying exclusively on a single node precludes meaningful network analysis, the broader Olink data was used purely as an exploratory screen based on nominal *p*-values, corroborated by subsequent RT-qPCR. Consequently, coupled with a modest sample size and an acute rat AMI model that inadequately mirrors the intricate pathophysiology of chronic IHD, the current study is fundamentally exploratory in nature, and claims regarding global functional myocardial recovery must be interpreted with caution.

In conclusion, within the constraints of this exploratory model, our data suggest that CSWT may facilitate angiogenesis and myocardial repair following AMI, potentially via the coordinated activation of CACs and the modulation of the S100A4/CSF2/FOXO1 protein network. This exploratory study provides preliminary mechanistic insights supporting further translational investigation of CSWT, framing it as a potential adjuvant therapy modulating the biology of myocardial repair.

## Data Availability

The original contributions presented in the study are included in the article/Supplementary Material, further inquiries can be directed to the corresponding author.
