# Supplementary material for: Extracorporeal cardiac shock wave therapy modulates post-infarction neovascularization via CAC-mediated pro-angiogenic effects and modulation of the S100A4/CSF2/FOXO1 protein network
Source: Front Cardiovasc Med. 2026 Jun 12;13:1839337. doi: 10.3389/fcvm.2026.1839337 (PMC13303029; doi:10.3389/fcvm.2026.1839337)
Supplement: Supplementary file 1 [file Table1.docx]

Supplementary Table 1: Differentially expressed proteins identified by Olink proteomics in three comparisons

[A = Control group;

B = Acute myocardial infarction (MI) group;

C = MI + cardiac shock wave therapy(MI+CSWT)group]

| Protein name | Log₂ fold change（A VS B） | Nominal p-value（A VS B） | Adjusted p-value（A VS B） | Log₂ fold change（A VS C） | Nominal p-value（A VS C） | Adjusted p-value（A VS C） | Log₂ fold change（B VS C） | Nominal p-value（B VS C） | Adjusted p-value（B VS C） |
| --- | --- | --- | --- | --- | --- | --- | --- | --- | --- |
| TGFBR3 | 0.87 | 0.00 | 0.03 | -0.33 | 0.01 | 0.09 | -1.21 | 0.00 | 0.03 |
| CLMP | 2.63 | 0.00 | 0.08 | 2.30 | 0.00 | 0.09 | -0.33 | 0.41 | 0.68 |
| PLA2G4A | 3.89 | 0.00 | 0.08 | 2.73 | 0.00 | 0.09 | -0.84 | 0.32 | 0.67 |
| DCTN2 | 1.80 | 0.00 | 0.08 | 0.96 | 0.27 | 0.56 | -0.84 | 0.32 | 0.67 |
| FSTL3 | 2.65 | 0.00 | 0.09 | 0.71 | 0.08 | 0.43 | -1.94 | 0.01 | 0.20 |
| FLRT2 | 3.86 | 0.01 | 0.11 | 3.14 | 0.00 | 0.09 | -0.73 | 0.18 | 0.66 |
| CA13 | 2.22 | 0.01 | 0.11 | 0.76 | 0.06 | 0.41 | -1.46 | 0.02 | 0.31 |
| WFIKKN2 | 1.19 | 0.01 | 0.12 | 0.60 | 0.07 | 0.41 | -0.59 | 0.07 | 0.49 |
| MAP2K6 | -0.89 | 0.02 | 0.18 | -0.18 | 0.24 | 0.55 | 0.72 | 0.02 | 0.31 |
| FST | 2.16 | 0.02 | 0.23 | 0.89 | 0.09 | 0.43 | -1.27 | 0.08 | 0.49 |
| CCL2 | 4.35 | 0.03 | 0.27 | 1.00 | 0.01 | 0.12 | -3.35 | 0.05 | 0.48 |
| EPCAM | 2.70 | 0.04 | 0.27 | 1.15 | 0.15 | 0.51 | -1.55 | 0.15 | 0.63 |
| SNAP29 | 0.84 | 0.05 | 0.27 | -0.07 | 0.77 | 0.86 | -0.91 | 0.04 | 0.42 |
| **S100A4** | **0.57** | **0.05** | **0.27** | **0.62** | **0.05** | **0.37** | **0.05** | **0.31** | **0.67** |
| IL10 | 1.61 | 0.05 | 0.27 | 0.36 | 0.52 | 0.74 | -1.24 | 0.02 | 0.31 |
| TGFB1 | 1.10 | 0.05 | 0.27 | 0.35 | 0.41 | 0.65 | -0.75 | 0.07 | 0.49 |
| ERBB4 | -1.27 | 0.05 | 0.27 | -0.70 | 0.04 | 0.36 | 0.57 | 0.22 | 0.67 |
| EDA2R | 1.45 | 0.06 | 0.27 | 0.35 | 0.23 | 0.55 | -1.10 | 0.08 | 0.49 |
| CASP3 | 2.81 | 0.06 | 0.27 | 1.31 | 0.10 | 0.43 | -1.50 | 0.18 | 0.66 |
| RGMA | -0.91 | 0.06 | 0.27 | -0.39 | 0.32 | 0.60 | 0.52 | 0.11 | 0.55 |
| ENO2 | 0.86 | 0.06 | 0.27 | 0.29 | 0.38 | 0.64 | -0.57 | 0.07 | 0.49 |
| TNFRSF12A | 0.66 | 0.08 | 0.33 | 0.11 | 0.82 | 0.88 | -0.55 | 0.33 | 0.67 |
| NADK | 1.45 | 0.10 | 0.39 | 0.13 | 0.62 | 0.80 | -1.33 | 0.12 | 0.55 |
| LGMN | 0.91 | 0.12 | 0.44 | 0.29 | 0.24 | 0.55 | -0.62 | 0.23 | 0.67 |
| ACVRL1 | 1.14 | 0.12 | 0.44 | 0.73 | 0.28 | 0.56 | -0.40 | 0.37 | 0.68 |
| TNNI3 | -2.09 | 0.13 | 0.45 | -0.23 | 0.61 | 0.80 | 1.86 | 0.16 | 0.63 |
| GHRL | -0.31 | 0.13 | 0.45 | 0.04 | 0.87 | 0.91 | 0.36 | 0.28 | 0.67 |
| CCL3 | 2.10 | 0.15 | 0.47 | 0.38 | 0.07 | 0.41 | -1.72 | 0.20 | 0.67 |
| IL1A | 0.73 | 0.15 | 0.47 | 0.59 | 0.23 | 0.55 | -0.14 | 0.62 | 0.85 |
| APBB1IP | 1.71 | 0.16 | 0.47 | 0.62 | 0.21 | 0.55 | -1.09 | 0.30 | 0.67 |
| NOTCH3 | -0.43 | 0.17 | 0.47 | 0.03 | 0.79 | 0.86 | 0.46 | 0.14 | 0.63 |
| **CSF2** | **-0.35** | **0.17** | **0.47** | **0.34** | **0.18** | **0.52** | **0.69** | **0.01** | **0.21** |
| GDNF | 0.92 | 0.17 | 0.47 | 0.69 | 0.11 | 0.43 | -0.23 | 0.68 | 0.91 |
| CCL20 | 0.57 | 0.18 | 0.50 | 0.41 | 0.41 | 0.65 | -0.16 | 0.70 | 0.91 |
| CCL5 | 1.21 | 0.19 | 0.51 | 0.17 | 0.80 | 0.86 | -1.04 | 0.19 | 0.66 |
| CXCL1 | 2.37 | 0.20 | 0.51 | 0.38 | 0.30 | 0.60 | -1.99 | 0.26 | 0.67 |
| IL1B | 0.47 | 0.21 | 0.51 | 0.40 | 0.23 | 0.55 | -0.07 | 0.82 | 0.92 |
| PARP1 | -0.51 | 0.24 | 0.54 | -0.43 | 0.12 | 0.43 | 0.08 | 0.83 | 0.92 |
| CNTN1 | -0.41 | 0.24 | 0.54 | -0.29 | 0.56 | 0.77 | 0.12 | 0.82 | 0.92 |
| FLI1 | 0.78 | 0.24 | 0.54 | -1.08 | 0.02 | 0.26 | -1.85 | 0.04 | 0.42 |
| TNFRSF11B | 0.30 | 0.25 | 0.56 | 0.65 | 0.16 | 0.52 | 0.35 | 0.38 | 0.68 |
| GCG | 0.65 | 0.25 | 0.56 | 0.85 | 0.17 | 0.52 | 0.19 | 0.50 | 0.75 |
| VEGFD | 0.34 | 0.30 | 0.64 | -0.03 | 0.93 | 0.96 | -1.34 | 0.40 | 0.68 |
| WISP1 | 1.72 | 0.31 | 0.64 | 0.38 | 0.18 | 0.52 | -1.34 | 0.40 | 0.68 |
| HGF | 1.19 | 0.33 | 0.66 | 0.28 | 0.38 | 0.64 | -0.91 | 0.44 | 0.69 |
| CYR61 | 0.98 | 0.34 | 0.66 | -0.26 | 0.36 | 0.63 | -1.24 | 0.26 | 0.67 |
| ITGB1BP2 | -0.68 | 0.34 | 0.66 | -0.08 | 0.47 | 0.70 | 0.60 | 0.39 | 0.68 |
| AXIN1 | -0.43 | 0.35 | 0.66 | -0.35 | 0.27 | 0.56 | 0.08 | 0.85 | 0.92 |
| AHR | 0.64 | 0.35 | 0.66 | 0.17 | 0.63 | 0.80 | -0.48 | 0.46 | 0.71 |
| QDPR | -0.38 | 0.36 | 0.66 | -0.23 | 0.12 | 0.43 | 0.15 | 0.69 | 0.91 |
| TNFSF12 | 0.22 | 0.37 | 0.67 | 0.05 | 0.73 | 0.86 | -0.17 | 0.53 | 0.79 |
| TGFA | 0.87 | 0.40 | 0.70 | -0.42 | 0.18 | 0.52 | -1.29 | 0.26 | 0.67 |
| CXCL9 | 2.36 | 0.41 | 0.70 | 2.64 | 0.36 | 0.63 | 0.28 | 0.42 | 0.69 |
| RIOX2 | -0.33 | 0.41 | 0.70 | -0.53 | 0.03 | 0.26 | -0.20 | 0.60 | 0.84 |
| PLXNA4 | 0.46 | 0.43 | 0.71 | 0.34 | 0.65 | 0.82 | -0.12 | 0.85 | 0.92 |
| CLSTN2 | -0.47 | 0.45 | 0.75 | 0.30 | 0.27 | 0.56 | 0.77 | 0.27 | 0.67 |
| DLL1 | -0.42 | 0.49 | 0.78 | -0.32 | 0.58 | 0.77 | 0.11 | 0.87 | 0.92 |
| FAS | -0.32 | 0.51 | 0.79 | 0.13 | 0.66 | 0.82 | 0.45 | 0.37 | 0.68 |
| IL17F | 0.17 | 0.52 | 0.79 | 0.18 | 0.49 | 0.71 | 0.00 | 0.98 | 0.99 |
| PDGFB | 0.21 | 0.53 | 0.79 | 0.16 | 0.68 | 0.83 | -0.05 | 0.87 | 0.92 |
| MATN2 | 0.71 | 0.53 | 0.79 | -0.35 | 0.49 | 0.71 | -1.06 | 0.39 | 0.68 |
| GFRA1 | -0.52 | 0.54 | 0.79 | 0.72 | 0.06 | 0.41 | 1.24 | 0.21 | 0.67 |
| LPL | 0.82 | 0.54 | 0.79 | 0.35 | 0.18 | 0.52 | -0.47 | 0.72 | 0.92 |
| **FOXO1** | **0.21** | **0.55** | **0.79** | **-1.35** | **0.02** | **0.26** | **-1.56** | **0.00** | **0.12** |
| TNF | 0.21 | 0.56 | 0.80 | 0.29 | 0.43 | 0.65 | 0.08 | 0.57 | 0.80 |
| PAK4 | -0.91 | 0.63 | 0.86 | -0.82 | 0.21 | 0.55 | 0.08 | 0.96 | 0.99 |
| NTF3 | -0.27 | 0.63 | 0.86 | -0.34 | 0.57 | 0.77 | -0.08 | 0.83 | 0.92 |
| CNTN4 | -0.22 | 0.64 | 0.86 | -0.09 | 0.72 | 0.85 | 0.13 | 0.78 | 0.92 |
| DDAH1 | -0.39 | 0.64 | 0.86 | 0.29 | 0.09 | 0.43 | 0.68 | 0.44 | 0.69 |
| IL5 | -0.23 | 0.68 | 0.89 | 0.44 | 0.43 | 0.65 | 0.67 | 0.30 | 0.67 |
| PPP1R2 | -0.18 | 0.70 | 0.90 | -0.38 | 0.30 | 0.60 | -0.20 | 0.71 | 0.91 |
| ADAM23 | 0.17 | 0.71 | 0.90 | 0.51 | 0.33 | 0.61 | 0.34 | 0.28 | 0.67 |
| YES1 | 0.08 | 0.74 | 0.93 | 0.36 | 0.00 | 0.09 | 0.28 | 0.30 | 0.67 |
| PRDX5 | -0.08 | 0.75 | 0.93 | -0.01 | 0.96 | 0.97 | 0.08 | 0.78 | 0.92 |
| DLK1 | -0.12 | 0.77 | 0.93 | 0.34 | 0.27 | 0.56 | 0.46 | 0.26 | 0.67 |
| IL23R | -0.20 | 0.78 | 0.93 | -0.23 | 0.54 | 0.75 | -0.03 | 0.97 | 0.99 |
| EPO | 0.09 | 0.78 | 0.93 | 0.04 | 0.70 | 0.84 | -0.05 | 0.88 | 0.92 |
| CANT1 | 0.07 | 0.82 | 0.95 | 0.10 | 0.76 | 0.86 | 0.04 | 0.88 | 0.92 |
| KITLG | -0.07 | 0.83 | 0.95 | 0.94 | 0.11 | 0.43 | 1.01 | 0.09 | 0.50 |
| SEZ6L2 | -0.21 | 0.84 | 0.95 | 0.89 | 0.04 | 0.36 | 1.10 | 0.34 | 0.68 |
| CDH6 | -0.06 | 0.87 | 0.95 | 0.12 | 0.77 | 0.86 | 0.18 | 0.56 | 0.80 |
| MIA | -0.05 | 0.87 | 0.95 | 0.30 | 0.41 | 0.65 | 0.35 | 0.37 | 0.68 |
| ITGB6 | -0.08 | 0.88 | 0.95 | 0.04 | 0.94 | 0.96 | 0.12 | 0.55 | 0.80 |
| PLIN1 | -0.29 | 0.88 | 0.95 | -1.49 | 0.41 | 0.65 | -1.20 | 0.39 | 0.68 |
| IGSF3 | 0.06 | 0.89 | 0.95 | -0.02 | 0.70 | 0.84 | -0.08 | 0.84 | 0.92 |
| TPP1 | 0.01 | 0.89 | 0.95 | 0.54 | 0.09 | 0.43 | 0.52 | 0.09 | 0.50 |
| IL6 | -0.03 | 0.93 | 0.98 | 0.36 | 0.12 | 0.44 | 0.39 | 0.26 | 0.67 |
| VSIG2 | 0.03 | 0.96 | 0.98 | 0.28 | 0.80 | 0.86 | 0.25 | 0.82 | 0.92 |
| TNR | 0.03 | 0.97 | 0.98 | -0.08 | 0.86 | 0.91 | -0.11 | 0.84 | 0.92 |
| CRIM1 | -0.01 | 0.97 | 0.98 | -0.01 | 0.97 | 0.97 | 0.00 | 0.99 | 0.99 |
| CPE | -0.01 | 0.98 | 0.98 | -0.11 | 0.36 | 0.63 | -0.10 | 0.81 | 0.92 |
| IL17A | 0.01 | 0.98 | 0.98 | 0.40 | 0.31 | 0.60 | 0.39 | 0.33 | 0.67 |
